# TGF-β in pancreatic cancer initiation and progression: two sides of the same coin

**DOI:** 10.1186/s13578-017-0168-0

**Published:** 2017-08-07

**Authors:** Wei Shen, Guo-qing Tao, Yu Zhang, Bing Cai, Jian Sun, Zhi-qiang Tian

**Affiliations:** Department of General Surgery, Wuxi People’s Hospital Affiliated Nanjing Medical University, 299 Qingyang Road, Wuxi, 214000 Jiangsu China

**Keywords:** Transforming growth factor-β, Pancreatic cancer, Tumor microenvironment

## Abstract

Pancreatic cancer is highly lethal malignant tumor with characterised rapid progression, invasiveness and resistance to radiochemotherapy. Transforming growth factor-β (TGF-β) signaling plays a dual role in both pro-tumorigenic and tumor suppressive of pancreatic cancer, depending on tumor stage and microenvironment. TGF-β signaling components alteration are common in pancreatic cancer, and its leading role in tumor formation and metastases has received increased attention. Many therapies have investigated to target TGF-β signaling in the preclinical and clinical setting. In this review, we highlight the dual roles of TGF-β and touch upon the perspectives on therapeutic target of TGF-β signaling in pancreatic cancer.

## Background

Pancreatic cancer is the fifth leading cause of death by cancer in the world [1]. The major histological subtype is pancreatic ductal adenocarcinoma, which comprises 90% of all pancreatic cancers. Pancreatic cancer is highly aggressive malignancy with an increasing incidence, which features rapid progression, invasiveness and resistance to radiochemotherapy [2]. At present the 5-year survival for pancreatic cancer is only 6% and the median survival from diagnosis is about 6 months [3]. Moreover, pancreatic cancer is expected to rise to the second leading cause of cancer-associated mortality by 2030 according to incidence’s prediction [4]. Current treatment choices available for pancreatic cancer show no significant improvement in overcoming the invasion and metastasis in the recent decades [5]. The key to improving is to control their local invasion, and distant metastasis, and these features underscore the pressing need to develop new therapeutic strategies specifically [6].

Transforming growth factor-β (TGF-β) plays an important role in regulating numerous normal cellular, physiological, and developmental processes. More evidence is emerging that TGF-β has a potential influence on the tumorigenic process. Deregulation of TGF-β signaling is involved in the pathophysiology of pancreatic cancer [7]. The insensitivity to growth inhibitory pathways is one of the hallmarks of cancer. Cancer genes consist of oncogenes and tumor-suppressor genes, but a growing number of them play a dual role and defy these categories. TGF-β signaling is one of the 12 core signaling pathways involved in pancreatic cancer. Mutation in at least one of the TGF-β signaling genes occurs in 100% of the pancreatic cancer. The action of TGF-β in pancreatic cancer is now attracting considerable attention. The role of TGF-β during pancreatic cancer initiation and progression is complex and somewhat paradoxical. TGF-β plays a tumor suppressor in early-stage pancreatic cancer by promoting apoptosis and inhibiting epithelial cell cycle progression, but plays a tumor promoter in late-stage by genomic instability, neoangiogenesis, immune evasion, cell motility, and metastasis [8].

In this review, we discuss recent insights into the regulation of TGF-β signaling and focus more on dual roles of TGF-β in pancreatic cancer. We also highlight knowledge on TGF-β signaling in cancer stem cells and tumor microenvironment of pancreatic cancer. We finally touch upon the perspectives on therapeutic target of TGF-β signaling in pancreatic cancer.

## TGF-β signaling pathways

**Fig. 1:**
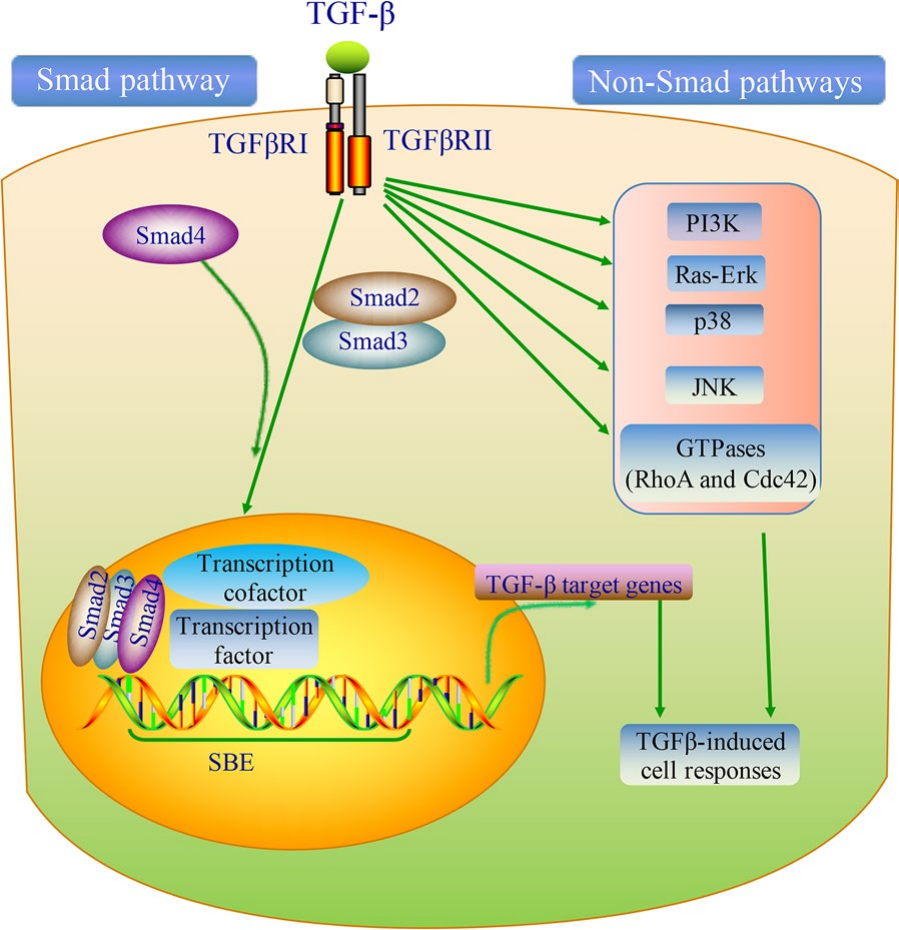
Overview of the TGF-β signaling pathway. TGF-β signaling is transduced through two pathways of Smad (canonical) and non-Smad (non-canonical). In Smad pathway, cell surface complexes of TGFβRI and TGFβRII phosphorylate upon TGF-β ligand binding and activate Smad2 and Smad3. Smad4 and activated Smad2/Smad3 form a Smads complex, and then interacts with other transcription factors to regulate transcription of target genes. TGF-β signaling also activates non-Smad pathways, including PI3K-Akt, Ras-Erk, p38, JNK, and GTPases

Recently, researchers are doing wide studies on the TGF-β signaling pathways. Three isoforms endow with the TGF-β of mammals, namely TGF-β1, TGF-β2, and TGF-β3 [9]. Each TGF-β is differentially expressed and activated during development and upon various cellular stresses [10]. Of these, TGF-β1 is the most abundant isoform in humans. In general, TGF-β1 expression is elevated by signals that promote cell growth and proliferation, whereas TGF-β2 and TGF-β3 are induced by differentiation and growth arrest signals. TGF-β signaling starts with activation and releasing of the TGFβ1. The TGF-β type I (TGFβRI) and type II receptors (TGFβRII) form heterotetrameric complexes at the cell surface and bind the dimeric ligands (Fig. 1). The functional receptor complex regulates the activation of downstream Smad and non‐Smad pathways. In Smad pathway, the activated TGFβRI/TGFβRII phosphorylates the Smad2 and Smad3 proteins, which modulate transcription in association with Smad4. The activated Smad complex translocates to the nucleus and binds to specific DNA sequence motifs called Smad-binding elements (SBEs). Upon binding, pSmad2/3-Smad4 complexes interact with additional transcriptional regulators to transactivate TGFβ-dependent genes (Fig. 1).

TGFβ-mediated tumor cell-autonomous and host-tumor interactions in cancer progression are also controlled by non-Smad pathways. Besides this ‘canonical’ signaling pathway, TGF-β signaling can also be transduced through the non-canonical Smad-independent pathways, including phosphatidylinositol-3 kinase (PI3K)/protein kinase B (AKT) pathway, JNK/p38 pathway, mitogen-activated protein kinase (MAPK) pathway, and Rho GTPases [11, 12] (Fig. 1). MED12, a transcriptional MEDIATOR complex protein, is mutated in cancers and found to interfere with maturation of TGF-βRII in the Golgi [13, 14]. MED12 loss therefore leads to activation of TGB-β signaling [15]. Consequently, TGF-β signaling causes activation of MEK/ERK signaling and restores the reduced MAPK pathway activation by tyrosine kinase inhibitors [15].

## TGF-β alterations in pancreatic cancer

Pancreatic cancer is a genetic disease characterized by somatic mutations of multiple genes [16]. The expression of TGF-β obviously increases in pancreatic cancer, and overexpression of TGF-β is associate with venous invasion, advanced tumor stages, progressive disease, shorter patient survival duration, and liver metastases [17–21]. Studies have shown that TGF-β signaling components often become genetically inactivated in pancreatic cancer and disabling TGF-β signaling may be a critical event in pancreatic cancer progression. Pancreatic cancer has detected loss of function or truncating mutations of TGFβRI, TGFβRII, Smad2, and Smad4 genes [22, 23]. Smad7, an inhibitory Smad family member, are proved overexpression and enhances tumorigenicity in human pancreatic cancer [24]. TGFβRII mutations are involved in 4–7% of pancreatic cancers [25–27], while mutations in TGFβRI are found in 2% of them [26–29]. Furthermore, 60% of pancreatic cancer is observed to lost 18q21 chromosome that harbors the Smad4 gene [28, 30]. Smad4 acts as a central mediator in the TGF-β signaling, and its inactivation is relatively specific for pancreatic cancer [11, 31–34]. KRAS mutation, which is necessary for carcinogenesis and subsequent cancer maintenance, is found in approximately 90% of all pancreatic ductal adenocarcinomas [35]. But, KRAS mutation alone is not sufficient for malignant transformation [36]. The data from whole-genome sequencing analyses demonstrated that the common co-mutations detected in pancreatic cancer are SMAD4, KRAS, MED12, TP53, and CDKN2A [37]. Mutations in tumor suppressors, such as SMAD4, SMAD4, and CDKN2A, are required for carcinogenesis in addition KRAS mutation [38]. So, losing the normal signaling of Smad4 may promote KRAS-driven malignant transformation of pancreatic duct cells [39].

## TGF-β in pancreatic cancer initiation and progression

TGF-β signaling function in pancreatic cancer appears complex and it is clearly evident that TGF-β acts in both an anti- and pro-tumorigenic activities. TGF-β exerts suppressive effects on tumor-promoting inflammation and on early stage of carcinogenesis, whereas during advance stage TGF-β acquires pro-oncogenic and pro-metastatic roles, which are associated with observable increase in the locally secreted TGF-β level [40–42].

### Tumor-suppressor role of TGF-β

TGF-β exhibits potent growth inhibitory effect in early stage of pancreatic cancer by promoting apoptosis and inhibiting cell cycle progression through G1 arrest [43]. Hezel et al. [44] found that TGF-β acts in a common tumor suppressor pathway whose pharmacologic inactivation promotes pancreatic cancer progression. TGF-β inhibits pancreatic cancer growth by decreasing VEGF and increasing thrombospondin-1, and perturbations of TGF-β signaling pathway during tumor progression relieves this inhibition [45]. Singh et al. [46] reported that TGF-β can inhibit pancreatic cancer cells growth in a p53-independent manner. Indeed, pancreatic cancer progression requires shutting down the tumor-suppressive effects of TGF-β signaling through mutation Smad transcription factors (Smad2, Smad4) [41].

### Tumor-promoter role of TGF-β

**Fig. 2:**
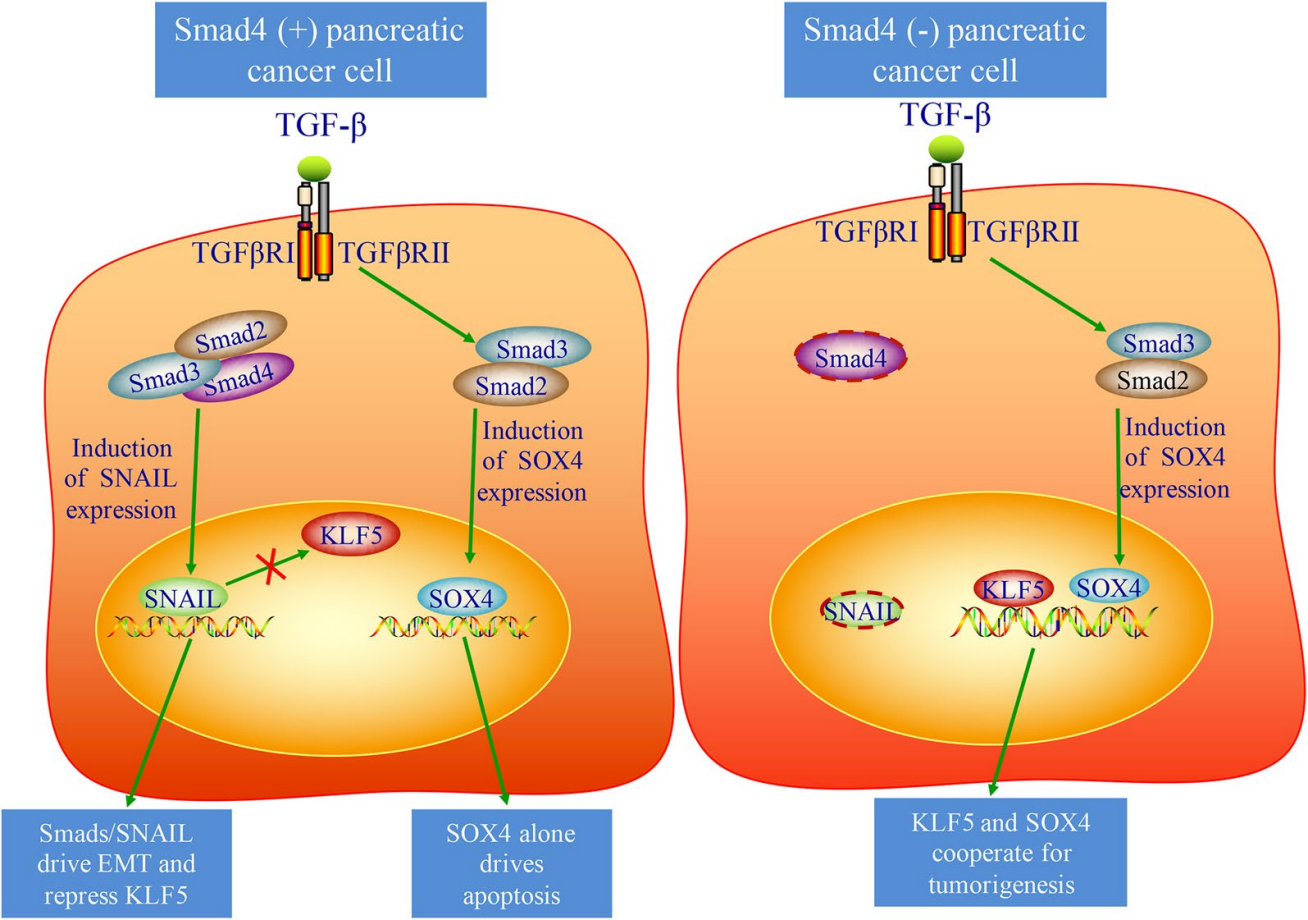
The dual role of TGF-β in pancreatic cancer cell. (*Left*) TGFβ-mediated epithelial–mesenchymal transition (EMT) and apoptosis in Smad4-positive pancreatic cancer cell. TGF-β signaling induces EMT by induction SNAIL and repression KLF5. Alternatively, TGF-β signaling induces apoptosis in KLF5 absence cell by SOX4 inducing transcription of pro-apoptotic genes. (*Right*) TGF-β promotes tumor progression in Smad4-negative pancreatic cancer cell. TGF-β signaling induces SOX4 induction mediated through Smad2/3, and then SOX4 and KLF5 cooperate for tumorigenesis

During advanced stage of carcinogenesis, TGF-β promotes invasion and metastasis of pancreatic cancer. TGF-β can promote stromal ‘‘activation’’, and induce angiogenesis, while attenuating a productive anti-tumor immune response [47, 48]. TGF-β ligands are commonly overexpressed in pancreatic cancer, and can promote epithelial-to-mesenchymal transition (EMT) and invasion in cell lines [49, 50]. TGF-β is one of the best known inducers of EMT-inducing transcription factors such as Snail, Slug, Twist, or Zeb1 [43]. However, David et al. demonstrate that TGF-β drives tumor suppression in pancreatic cancer cells by promoting EMT-linked remodeling of the transcription factor landscape, which converts TGFβ-induced Sox4 from an enforcer of tumorigenesis in the epithelial state into a promoter of apoptosis after EMT [51]. TGF-β induces an EMT generally considered as a pro-tumorigenic event. However, in TGFβ-sensitive pancreatic adenocarcinoma cells, EMT becomes lethal by converting TGFβ-induced Sox4 from an enforcer of tumorigenesis into a promoter of apoptosis [51] (Fig. 2). This study provides elegant mechanistic data to elucidate the dichotomous effects of TGF-β on pancreatic cancer cells [51] (Fig. 2).

## TGF-β actions in cancer stem cells

Cancer stem cells, a subpopulation of cancer cells with stem cell characteristics, are widely believed responsible for tumor carcinogenesis, progression and recurrence [52]. Recent studies have demonstrated that cancer stem cells stay in quiescent status and resist to traditional chemo-therapy and radio-therapy [53]. Researchers isolated cancer stem cells from pancreatic cancer which were characterized with self-renewal, highly tumorigenic, and more differentiated progenies [54, 55]. Cancer stem cells are the root of cancer which cannot be killed by traditional methods. So, developing novel drugs or approaches to radically eliminate the origin of tumor cells will bring great effect on cancer therapy. The signal pathways contributing to self-renew is an important research direction of exploring the targeting drugs. Research increasingly suggests that TGF-β plays an important role in the occurrence and development of pancreatic cancer stem cells. TGF-β signaling has been confirmed more widely role in the maintenance of pancreatic stem cells [56]. TGF-β signaling through Activin/Nodal activation is required for self-renewal and tumorigenicity of cancer stem cells in pancreatic cancer [57].

## TGF-β actions in tumor microenvironment

Pancreatic cancer displays greater prominent desmoplastic stromal reaction, though the cancer itself is the epithelial component [58]. The expression of TGF-β shows high level in pancreatic cancer tissue [59]. The microenvironment of pancreatic cancer features a pronounced stromal reaction composed of collagen-rich extracellular matrix, pancreatic stellate cells, and inflammatory cells [60, 61]. Tumor microenvironment plays a significant role in tumor initiation and development, and it can influence the interaction between pancreatic cancer cells and TGF-β [62]. Pancreatic stellate cells are responsible for excess extracellular matrix production in pancreatic cancer. TGF-β, as a potent activator, mediates the interaction between pancreatic stellate cells and cancer cells [63]. Growth factors produced and released by stroma to pancreatic cancer cells result with the reactive stroma [64]. These stromal elements in addition to TGF-β signaling participation in autocrine and paracrine produce a modified extracellular matrix that can accelerates growth and metastasis of pancreatic cancer cells [64, 65]. In sum, TGF-β has the dual role at the microenvironment level of pancreatic cancer. TGF-β is initially utilized to prevent occurrence and proliferation of pancreatic cancer in precancerous and early stage, but it is ultimately used to promote pancreatic cancer progression in advanced stage of carcinogenesis.

## Therapeutic perspectives

TGF-β signaling is considered a prominent treatment target for pancreatic cancer in oncology [66]. Several relevant therapeutic approaches have been investigated in the preclinical and clinical setting and have shown efficacy [40, 67–71]. Ligand, ligand-receptor binding and intracellular signal transduction are the three levels of the therapeutic strategies to disrupt TGF-β signaling, such as TGF-β antisense RNA molecules, TGF-β blocking antibodies, neutralizing antibodies to the TGFβRII, and TGFβRI kinase small molecule inhibitors [40, 69, 70].

### Therapeutic strategy on the ligand level

RNA interference (RNAi) has been applied to restrain the synthesis of TGF-β by regulation of TGFβ-coding genes expression. The short interfering RNA (siRNA) and the micro interfering RNA (miRNA) are mainly two types of antisense RNA molecules. Trabedersen (AP 12009), a TGF-β2 antisense RNA molecule, significantly reduced tumor growth, angiogenesis and lymph node metastasis in a metastatic pancreatic cancer mouse model [68, 72, 73].

### Therapeutic strategy on the ligand-receptor interaction level

Natural TGF-β inhibitors, monoclonal blocking antibodies and soluble TGF-β receptors are mainly compounds of intervention on the ligand–receptor level. A soluble TGFβRII protein that blocks cellular responsiveness to TGF-β1 could reduce pancreatic cancer cell metastasis by the expression decrease of metastasis-associated genes in an orthotopic mouse model [67, 71]. Murakami et al. [74] described the efficacy of SB431542, a TGFβRI inhibitor, in a human pancreatic-cancer orthotopic mouse model by color-coded intravital imaging. The result of study demonstrated that color-coded intravital imaging readily detect the selective anti-stromal-cell targeting of SB431542.

### Therapeutic strategy on the intracellular signaling level

Most of inhibitors on the intracellular signaling level target the kinase of TGF-β receptors. But others are peptide aptamers targeting Smads interaction with TGF-β receptors. SD-208, an inhibitor of TGFβRI kinase, reduced pancreatic cancer growth and metastasis in vivo and reduced fibrosis in the tumor microenvironment [72, 75]. SD-093, a selective inhibitor of TGFβRI kinase, strongly reduced the motility and invasiveness of the pancreatic cancer cells in vitro [76, 77]. LY2109761, a dual inhibitor of TGFβRI/II kinase, significantly reduces the tumor burden, abdominal metastases, and improves survival of metastatic pancreatic cancer in a murine model [68]. Galunisertib (LY2157299), an inhibitor of TGFβRI kinase, has been demonstrated an acceptable tolerability and safety profile in Japanese patients with advanced pancreatic and lung cancers in a phase 1 clinical study [78]. However, Oyanagi et al. [79] reported that galunisertib (LY2157299) can promote the invasion in collagen matrix of pancreatic carcinoma cells through hepatocyte growth factor produced by fibroblast. Gore et al. [80] reported that combinatorial targeting of TGFβRI with LY2157299 and EGFR/HER2 with lapatinib suppresses lymphangiogenesis and metastasis in a syngeneic orthotopic pancreatic cancer model. Therefore, under some pathological conditions, the inhibitors TGF-β signaling may contribute to development of cancer [79]. To face the metastasis dissemination challenge of cancer patients, the therapeutic strategy of intervention TGF-β signaling has been approached over the years. The inhibitors of TGF-β signaling have been shown effective in a number of studies of pancreatic cancer patients. Yet, developmental work requires to further efforts in novel type of inhibitors,e.g. substrate-mimicking drugs.

## Conclusions

TGF-β signaling has kind of a dual role of promotion and inhibition in pancreatic cancer depending on different cancer stage and microenvironment. The alteration of TGF-β signaling components in pancreatic cancer is common and pronounced, and its leading role in cancer formation and metastases is arousing more attention. TGF-β also conducts a pivotal role of caner stem cells and tumor microenvironment in pancreatic cancer. TGF-β signaling targeted therapies have been investigated in the preclinical and clinical setting and have shown efficacy in pancreatic cancer. This novel strategy may be lead to the identification of improved outcomes for lethal pancreatic cancer.

## Abbreviations

TGF-β: transforming growth factor-β
EMT: epithelial-to-mesenchymal transition
